# Role and mechanism of gut microbiota in regulating interferon-mediated programmed cell death in colorectal cancer

**DOI:** 10.3389/fimmu.2025.1724908

**Published:** 2026-01-12

**Authors:** Qipeng Yao, Shiyin Chen, Weiwei Qian, Chao Yang, Junxian Li

**Affiliations:** 1 Department of Chinese Medicine, Sichuan Provincial People’s Hospital, School of Medicine, University of Electronic Science and Technology of China, Chengdu, China; 2 Emergency Department, Chengdu Shangjin Nanfu Hospital, Chengdu, Sichuan, China; 3 Department of Traditional Chinese Medicine Surgery, Sichuan Provincial People’s Hospital, School of Medicine, University of Electronic Science and Technology of China, Chengdu, China

**Keywords:** colorectal cancer, gut microbiota, immune microenvironment, interferon, programmed cell death

## Abstract

Colorectal cancer (CRC) is a highly prevalent and lethal malignancy worldwide, whose development is closely associated with gut microbiota dysbiosis and immune microenvironment imbalance. Interferons (IFNs) serve not only as pivotal cytokines bridging innate and adaptive immunity but also induce multiple forms of programmed cell death (PCD), playing a crucial role in antitumor immunity. This narrative review examines the core mechanisms of the gut microbiota-IFNs-programmed cell death axis within the CRC immune microenvironment. As upstream regulators, gut microbiota profoundly influence the production and function of type I, II, and III interferons through metabolic products and microbial-associated molecular patterns (MAMPs). Conversely, IFNs, serving as the pivotal link between innate and adaptive immunity, directly participate in tumor immune surveillance while also determining tumor cell fate by finely regulating PCD pathways such as apoptosis, autophagy, pyroptosis, and ferroptosis. In the CRC context, protective microbiota enhances IFN signaling and promote immunogenic PCD, activating effective antitumor immunity. Conversely, carcinogenic microbiota suppresses IFN responses, disrupt immune surveillance, and drive immune evasion and drug resistance. In-depth investigation of the mechanisms by which gut microbiota modulate interferon-mediated programmed cell death in CRC not only offers new insights into CRC immune evasion but also provides a theoretical foundation for developing combined immunotherapy strategies based on microbiota intervention, targeting IFN pathways, or regulating PCD patterns.

## 1 Introduction

Colorectal cancer (CRC) ranks as the third most prevalent and second deadliest malignant tumor globally. According to GLOBOCAN 2020 statistics, annual new cases and deaths amount to approximately 1.9 million and 900,000, respectively (1). With population aging and lifestyle changes, its incidence continues to rise in developing countries, posing a significant public health burden (2). The development of CRC exhibits high heterogeneity, involving multifactorial influences such as genetic mutations, environmental factors, immune dysregulation, metabolic disorders, and gut microbiota imbalance (1, 3–5). However, its precise pathogenesis remains incompletely elucidated. Notably, the gut microbiota—as a core microecosystem maintaining intestinal mucosal barriers, immune homeostasis, and metabolic equilibrium—has gained increasing prominence in CRC research (6–8). Research indicates that dysbiosis can promote polyp formation and tumor progression through mechanisms such as disrupting mucosal integrity, inducing metabolic abnormalities, and producing carcinogens (9–12). Furthermore, the gut microbiota composition of CRC patients exhibits significant differences compared to healthy individuals. Functional experiments and animal models have further confirmed that multiple bacteria play key roles in CRC development (13, 14). Conversely, probiotics can reduce CRC risk by regulating microbial balance and inhibiting pathogenic bacterial proliferation (15, 16). Therefore, elucidating the specific mechanisms of gut microbiota in CRC development and immune regulation is crucial for uncovering the disease’s etiology, developing novel biomarkers, and establishing microbiome-based intervention strategies.

Interferons (IFNs) serve as core cytokines in both innate and adaptive immunity, with functions spanning antiviral defense, tumor immune surveillance, and inflammatory response regulation (17–19). Based on structural and receptor differences, IFNs are primarily classified into Type I, Type II, and Type III. They activate signaling pathways such as JAK–STAT to induce expression of downstream interferon-stimulated genes (ISGs), thereby achieving broad immunomodulatory effects. Beyond their classic antiviral and antitumor roles, IFNs also participate in shaping the immune microenvironment and eliminating tumor cells by regulating multiple forms of programmed cell death (PCD), including apoptosis, autophagy, pyroptosis, and ferroptosis (20). Recent studies reveal a close regulatory relationship between the IFN signaling pathway and the gut microbiota: microbial composition and its metabolites significantly influence IFN production and function; conversely, IFNs can also affect microbial structure and the tumor immune microenvironment by inducing PCD and exerting immunomodulatory effects (21–23).

Exploring the interactions among gut microbiota, interferons, and programmed cell death, along with their regulatory mechanisms in the CRC immune microenvironment, holds significant scientific and clinical value. This paper aims to present a narrative review of the IFN signaling pathway and PCD mechanisms, elucidate the interactive relationship between gut microbiota and IFN, and focus on analyzing the action mechanisms of this regulatory axis within the CRC immune microenvironment. This endeavor seeks to provide new theoretical foundations and potential targets for immunotherapy strategies in colorectal cancer.

## 2 Regulatory role of gut microbiota in the immune microenvironment of CRC

The development of colorectal cancer is influenced not only by host genetic mutations and the infiltration status of immune cells, but also profoundly regulated by the homeostasis and dysbiosis of the gut microbiota. A growing body of research indicates that gut microbiota profoundly shapes the tumor immune microenvironment through metabolic products, signaling molecules, and interactions with immune cells. This bidirectional regulatory relationship between microbiota and the immune microenvironment not only reveals key mechanisms underlying CRC development.

### 2.1 Gut microbiota

The human microbiome constitutes a dynamic ecosystem comprising approximately 10^14^ microorganisms spanning around 3,000 species, including bacteria, fungi, and viruses. Significant variations exist in the quantity and abundance of different microbial types, with high heterogeneity in composition among individuals. Bacteria dominate quantitatively and exert profound effects on host health (24, 25). This ecosystem plays a crucial role in maintaining physiological homeostasis and functional balance. The vast majority of microorganisms colonize the gastrointestinal tract, with the highest density found in the colon, forming the extensively studied “gut microbiota” (26).

Among the diverse array of bacteria, the phyla Firmicutes, Bacteroidetes, Actinobacteria, and Proteobacteria represent the major dominant groups (27, 28). Research indicates that the gut microbiota participates in regulating multiple physiological functions of the host: in digestion and nutrient metabolism, they assist in breaking down dietary components difficult for the host to digest (such as cellulose), produce short-chain fatty acids (SCFAs) through fermentation to provide energy for the host, and participate in vitamin synthesis and absorption (29, 30). Regarding immune regulation, gut microbiota is crucial for the maturation and development of the immune system (31, 32). Disruption of the host-microbiota balance leads to dysbiosis, manifested by reduced microbial diversity, imbalanced dominant species ratios, altered metabolic profiles, and abnormal secretion of vesicles and signaling molecules. Dysbiosis can impair intestinal mucosal barrier function, trigger chronic inflammatory responses, disrupt immune tolerance, and facilitate pathogenic bacterial colonization, thereby affecting host metabolic homeostasis. These alterations are closely associated with the development of diseases and physiological dysregulation, including inflammatory bowel disease, metabolic syndrome, neurological disorders, and various cancers (33, 34).

### 2.2 Colorectal cancer and gut microbiota

The immune microenvironment of colorectal cancer constitutes a highly dynamic ecosystem, where the types, density, and spatial distribution of immune cells exert decisive influence on tumor progression and patient prognosis. The quantity and localization of tumor-infiltrating lymphocytes (TILs), such as CD8^+^ T cells and CD45RO^+^ memory T cells, serve as critical survival predictors. The resulting “immunoscore” has emerged as an independent prognostic tool beyond TNM staging, driving the development of TNM-Immune integrated staging (35–37). Effector T cells, NK cells, and M1 macrophages suppress tumors through cytotoxic actions. The consensus molecular subtyping (CMS) of CRC further reveals its immune heterogeneity: CMS1 represents an immune-activated subtype sensitive to immunotherapy, while CMS4 is enriched with immunosuppressive cells and carries a poorer prognosis (3). Immune checkpoint inhibitors demonstrate significant efficacy in dMMR/MSI-H patients, highlighting the critical regulatory role of immune infiltration status in treatment response (38).

In recent years, research has increasingly revealed that the gut microbiota plays a crucial role in shaping the immune microenvironment of CRC (39). Under steady-state conditions, the gut microbiota maintains intestinal homeostasis by preserving the mucosal barrier, competitively inhibiting pathogenic bacteria, producing metabolites, and regulating mucosal immunity. However, reduced microbial diversity, enrichment of pro-carcinogenic strains, or depletion of beneficial bacteria disrupts this equilibrium, promoting precancerous lesions and tumorigenesis (8, 40). The microbiota of CRC patients often exhibits a pattern of “oncogenic bacteria enrichment—beneficial bacteria depletion”: significant increases in enterotoxigenic Bacteroides fragilis (ETBF), Fusobacterium nucleatum, and pks island-carrying Escherichia coli, alongside marked reductions in butyrate-producing bacteria (41, 42). Specifically, ETBF secretes BFT toxin, which disrupts the epithelial barrier, activates STAT3 and Wnt signaling, and promotes IL-17/IL-23 inflammatory axis activation, thereby accelerating tumorigenesis (43). F. nucleatum activates the β-catenin pathway via its adhesion protein FadA to upregulate oncogenes, and suppresses NK and T cell function by binding TIGIT through its Fap2 protein, thereby achieving immune evasion (44, 45). pks^+^ E. coli synthesizes colibactin, directly causing DNA damage and accumulating mutations (46–48). Furthermore, certain microbial metabolites like secondary bile acids (deoxycholic acid, DCA) induce DNA damage and accelerate carcinogenesis (49). Conversely, beneficial bacteria and their metabolites exert protective effects in CRC prevention and control. SCFAs particularly butyrate, serve as both an energy substrate for epithelial cells and a histone deacetylase inhibitor that promotes cancer cell apoptosis. They also enhance CD8^+^ T cell and memory T cell function, thereby improving antitumor immunity and mitigating the proinflammatory environment (50, 51). Probiotics such as *Lactobacillus*, *Bifidobacterium*, and *Faecalibacterium prausnitzii* have also been shown to reduce CRC risk by maintaining intestinal barrier integrity, secreting anti-inflammatory metabolites, and modulating immune responses (52, 53) (**Table 1**).

**Table 1:** Gut microbiota-induced colon cancer.

Bacteria/Metabolites	Effectors	Mechanisms	References
*Enterotoxigenic Bacteroides fragilis*	BFT	Activate the β-catenin and STAT3 pathways, increasing the expression of COX-2 and NF-κB.	(43)
*Fusobacterium nucleatum*	FadA, Fap2	Modulate the E-cadherin/β-catenin pathway to achieve immune evasion.	(44, 45)
*Escherichia coli*	Colibactin, cyclolethal distending toxins (CDTs)	Cause DNA double-strand breaks	(46–48)
*Enterococcus faecalis*	Metalloprotease	Damage DNA by generating reactive oxygen species (ROS) and extracellular superoxide	(54, 55)
*Peptostreptococcus anaerobius*	PCWBR2	TLR-2 and TLR-4 on colonic cells interact, inducing ROS formation.	(56)
*Streptococcus bovis/gallolyticus*	Pil3 pilus	Increase β-catenin promotes inflammation and cell proliferation.	(20)

In the development of CRC, the interaction between the gut microbiota and the host immune system forms a complex regulatory network. Specific microbial communities can drive tumor progression by inducing immune suppression and chronic inflammation, while beneficial microbiota exert protective effects by activating anti-tumor immunity. The underlying mechanisms of this microbiota-immune interaction are closely linked to the regulation of core immune signaling pathways, such as those involving interferons.

## 3 Interferon signaling pathways and programmed cell death mechanisms

### 3.1 Fundamental biological functions of interferons

Interferons are a class of cytokines produced by host cells in response to stimuli such as viral infection, exhibiting antiviral, immunomodulatory, and antitumor functions. Based on differences in expression sources, target cell specificity, and immunoregulatory functions, they are primarily classified into Type I, Type II, and Type III. These interferons synergistically activate distinct signaling pathways and immune mechanisms, playing a pivotal regulatory role in bridging innate and adaptive immunity. They constitute a critical safeguard for the body against diverse pathogen infections and for maintaining immune homeostasis (19, 57, 58).

#### 3.1.1 Type I interferons

Type I interferons (IFN-Is), first identified in 1957, have long been recognized as essential antiviral mediators of the innate immune response (59, 60). IFN-I family comprises 17 functional members in humans and 18 in mice and is produced primarily by dendritic cells, epithelial cells, and macrophages upon sensing pathogen-derived nucleic acids or damage-associated signals (19, 61, 62). The IFNAR receptor complex is broadly expressed on nucleated cells, enabling IFN-I to induce a wide array of interferon-stimulated genes (ISGs) through the JAK–STAT signaling pathway (17, 63), while negative regulators such as SOCS maintain signaling homeostasis and prevent immunopathology (64, 65). Functionally, IFN-I enhances NK cell cytotoxicity and promotes dendritic cell maturation, as well as T and B cell responses (66–69). However, persistent or excessive IFN-I signaling can induce PD-L1 upregulation, T-cell exhaustion, and chronic inflammation, thereby impairing antiviral or antitumor immunity (70–74). Therefore, precise regulation of IFN-I signaling is crucial for maintaining immune homeostasis and achieving antitumor therapeutic outcomes.

Recent studies have demonstrated that the gut microbiota enhances host IFN-I responses through multiple innate immune pathways. Microbiota-derived LPS, CpG-DNA, and flagellin can activate TLR4, TLR9, and TLR5, respectively, thereby inducing IFN-I production via IRF3/7 signaling (75–77). In addition, bacterial DNA or outer membrane vesicles (OMVs) can be internalized by host cells and trigger the cGAS–STING pathway, further amplifying IFN-I signaling (78). Moreover, short-chain fatty acid (SCFA)–producing *Blautia* species, as well as probiotic strains such as *Lactobacillus rhamnosus* GG, can promote IFN-I production through the MAVS–IRF3–IFNAR axis, thereby supporting basal antiviral immunity (79, 80).

#### 3.1.2 Type II interferon

Type II interferon (IFN-γ) is the sole member of the type II interferon family and is primarily produced by activated NK cells and Th1/CD8^+^ T cells in response to antigenic or cytokine stimulation such as IL-12 and IL-18 (81, 82). Upon binding to its receptor IFNGR1/2, IFN-γ activates the JAK1/JAK2–STAT1 signaling axis, leading to γ-activated factor (GAF)–dependent transcription of interferon-responsive genes (83, 84). Functionally, IFN-γ enhances the antimicrobial activity of macrophages, upregulates MHC-I/II to improve antigen presentation, promotes Th1 and CTL-mediated cellular immunity, and suppresses Th2 and Treg responses (81–83, 85). Within the tumor microenvironment, IFN-γ facilitates immune cell infiltration and antigen visibility, contributing to tumor immunosurveillance and growth control (86, 87).

Gut microbiota exerts profound regulatory effects on the induction and effector functions of IFN-γ through multiple layers of immune modulation. First, microbe-associated molecular patterns such as LPS, CpG-DNA, and flagellin activate TLR4, TLR9, and TLR5 on dendritic cells and macrophages, inducing Th1-polarizing cytokines including IL-12 and IL-18 and thereby directly promoting IFN-γ production by NK cells and Th1/CD8^+^ T cells (88–91). Second, bacterial DNA or outer membrane vesicles (OMVs) entering host cells can activate the cGAS–STING pathway, enhancing antigen presentation and co-stimulatory molecule expression in dendritic cells, which raises the threshold for T-cell IFN-γ induction (92, 93). In addition, microbial metabolites critically shape IFN-γ programs: short-chain fatty acids (SCFAs) modulate IL-12 production by dendritic cells and promote CD8^+^ T-cell effector differentiation (94, 95), and indole derivatives regulate mucosal immune homeostasis through AHR signaling, thereby influencing IFN-γ expression in Th1 and NK cells (96, 97). Certain probiotics, including *Bifidobacterium* and *Lactobacillus* species, have also been shown to enhance Th1 polarization and NK-cell activation, further augmenting IFN-γ responses (98).

#### 3.1.3 Type III interferons

Type III interferon (IFN-λ) encompass human IFN-λ1 through λ4, first reported by Sheppard and Kotenko et al. in 2003 (99, 100). Structurally similar to the IL-10 family, this group exhibits typical interferon functions. Their receptor complex comprises IFNLR1 (interferon lambda receptor 1) and IL-10Rβ (IL-10 receptor subunit beta), primarily expressed on epithelial cells and some neutrophils (19, 101). They demonstrate marked tissue specificity.

The signaling mechanism activated by IFN-λ resembles that of IFN-I, also promoting STAT1/2 phosphorylation through the JAK–STAT pathway and forming the ISGF3 complex to drive ISG expression (102). Unlike systemically acting IFN-I, IFN-III primarily induce a localized, mild, and persistent antiviral state in mucosal epithelium, avoiding systemic inflammation and immunopathology (103–105). Consequently, IFN-λ possesses unique advantages in maintaining barrier immune homeostasis, restricting viral mucosal replication, and protecting tissues from inflammatory damage, playing a critical role in mucosal defense, particularly in the respiratory, digestive, and reproductive tracts (106).

### 3.2 Interferon regulation of programmed cell death

Programmed cell death is a genetically regulated, proactive form of cell death executed according to specific protocols. Unlike passive necrosis, it plays a critical role in development, homeostasis maintenance, and immune responses. Based on molecular mechanisms and morphological characteristics, PCD primarily encompasses apoptosis, autophagy, pyroptosis, and ferroptosis. Interferons exert fine-tuned regulatory effects across multiple PCD pathways. By modulating interactions between death signals and immune effector cells, they shape the immune microenvironment and determine cellular fate.

#### 3.2.1 Apoptosis

Apoptosis is a non-inflammatory, energy-dependent form of cell death that is crucial for development, immune homeostasis, and tumor defense. Characterized by cell shrinkage, membrane vesiculation, chromatin condensation, and the formation of apoptotic bodies, it maintains tissue homeostasis by eliminating abnormal or damaged cells. Imbalances in apoptosis are closely associated with tumors, neurodegenerative diseases, and autoimmune disorders (107, 108).

At the molecular level, apoptosis is mediated by the extrinsic death receptor pathway and the intrinsic mitochondrial pathway. The extrinsic pathway activates caspase-8 via Fas or TNFR; the intrinsic pathway induces mitochondrial release of cytochrome c and activates caspase-9 following stress or DNA damage, ultimately converging on effector caspase-3. The Bcl-2 family regulates this process: Bcl-2 and Bcl-xL inhibit apoptosis, while Bax, Bak, and Bid promote it (109–112). Anti-tumor strategies targeting apoptosis have garnered significant attention. Compounds such as the small molecule YLT322, red amine acid, resveratrol, and platycodin D can induce apoptosis via the mitochondrial pathway and enhance immune responses (113–116).

Interferons promote apoptosis by coupling exogenous and endogenous signals, playing a pivotal role in tumor immunity. IFNs upregulate death ligands such as TRAIL (tumor necrosis factor–related apoptosis-inducing ligand), FasL (Fas ligand), and TNF-α (Tumor necrosis factor alpha), activating the caspase-8 cascade; caspase-8 further cleaves Bid to generate tBid, enhancing mitochondrial membrane permeability and amplifying apoptotic signals (117–121). Concurrently, IFNs upregulate pro-apoptotic factors STAT1, IRF1, Bim, and XAF1 while suppressing Bcl-2 expression, heightening cellular sensitivity to apoptotic stimuli (117, 118, 122). Notably, IFN-γ induces iNOS expression and synergistically activates BAX/BAK with caspase-8, intensifying apoptotic effects in tumor cells (120).

#### 3.2.2 Autophagy

Autophagy is an intracellular degradation pathway unique to eukaryotic cells. It maintains cellular homeostasis and energy balance by forming autophagosomes that engulf damaged organelles or proteins, which are then degraded upon fusion with lysosomes (123, 124). Autophagy plays crucial roles in responding to nutrient deprivation, oxidative stress, pathogen invasion, and antigen presentation (123, 125). Based on substrate delivery mechanisms, autophagy is categorized into macroautophagy, microautophagy, and chaperone-mediated autophagy (CMA), which collaborate to sustain cell survival and stress adaptation (126).

Interferons induce autophagy and modulate immune responses through multiple signaling pathways. IFN-γ activates autophagy by upregulating autophagy genes such as Beclin1 and ATG5 via IRF1, promoting ROS accumulation, and activating the AMP-activated protein kinase (AMPK)–mechanistic target of rapamycin (mTOR) inhibitory axis. It also synergistically mediates tumor cell death with apoptosis (127, 128). IFN-I regulates Beclin1 and microtubule-associated protein 1 light chain 3 beta (LC3B) expression via the JAK–STAT pathway, inducing autophagy in a dose-dependent manner across multiple models including hepatocellular carcinoma (129), and can influence autophagy protein stability through miRNA-mediated post-transcriptional regulation (130). IFN-III similarly promote autophagy and lysosome formation in a dose-dependent manner in osteosarcoma cells, inhibiting cell invasion and metastasis (131). Furthermore, IFNs regulate autophagosome formation through the stimulator of interferon genes (STING)–TANK-binding kinase 1 (TBK1) pathway and ubiquitination of the autophagy receptor p62/SQSTM1 (132). Collectively, interferon-induced autophagy not only participates in cell death but also maintains metabolic and immune homeostasis in antiviral defense and antitumor immunity.

#### 3.2.3 Pyroptosis

Pyroptosis is a form of inflammasome-dependent programmed cell death characterized by distinct proinflammatory features (133). Its hallmarks include cellular swelling, membrane rupture, leakage of cellular contents, and release of IL-1β and IL-18. Pyroptosis serves an immune defense function by eliminating infected cells and activating inflammation, but uncontrolled pyroptosis can cause tissue damage. The canonical pathway involves pathogen-associated molecular patterns (PAMPs) or damage-associated molecular patterns (DAMPs) activating receptors such as NLRP3, NLRC4, or AIM2, leading to inflammasome assembly and caspase-1 activation, which cleaves gasdermin D (GSDMD) and releases cytokines. In the non-canonical pathway, caspase-4/5 (human) or caspase-11 (mouse) directly recognizes intracellular lipopolysaccharide (LPS), cleaves GSDMD to form membrane pores, and further activates NLRP3 to amplify the response (133–137).

Interferons regulate pyroptosis through multiple mechanisms. IFN-I induce the expression of ZBP1 (Z-DNA-binding protein 1), MLKL (mixed lineage kinase domain-like protein), caspase-11, and GSDMD, thereby promoting NLRP3 activation and GSDMD-dependent pyroptosis (138). In severe acute pancreatitis, mitochondrial DNA activates NLRP3 via the cGAS–STING (cyclic GMP–AMP synthase–stimulator of interferon genes) pathway, exacerbating macrophage pyroptosis and inflammatory responses (139). IFN-III exert negative regulation by upregulating ZBP1 and activating the caspase-8/GSDMC pathway to induce intestinal epithelial pyroptosis, thereby delaying epithelial repair after colitis or radiation injury. This pathway is highly activated in inflammatory bowel disease (119). In tumor immunity, interferons can induce pyroptosis mediated by caspase-1 or caspase-4/5/11 through NLRP3 or cGAS–STING pathways, releasing IL-1β and IL-18 to promote immune cell infiltration and antitumor responses. Studies demonstrate that IFN-γ induces caspase-4 expression in lung adenocarcinoma cells, triggering pyroptosis under high-concentration stimulation. Inhibiting the negative regulator USP18 (ubiquitin-specific peptidase 18) enhances STAT2 activity and induces immunogenic pyroptosis, thereby amplifying antitumor effects (140, 141). Furthermore, in renal cell carcinoma, STING deletion or degradation via the PROTAC agent SP23 activates GSDMD-dependent pyroptosis through the PERK/eIF2α/ATF4/CHOP–caspase-8 axis, enhancing CD4^+^/CD8^+^ T cell infiltration and thereby strengthening antitumor immunity (142). These findings indicate that interferon regulates immunogenic cell death via the pyroptotic pathway in both anti-infection and tumor immunity.

#### 3.2.4 Ferroptosis

Ferroptosis is an iron-dependent, lipid peroxidation-driven form of cell death distinct from apoptosis and necrosis. Its hallmark features include excessive intracellular free iron accumulation, markedly elevated reactive ROS levels, massive accumulation of membrane lipid peroxides, and dysfunction of the glutathione peroxidase 4 (GPX4) antioxidant defense system (143–145). Its core mechanism involves glutathione (GSH) depletion and loss of GPX4 activity, leading to the oxidation of polyunsaturated fatty acids (PUFAs) into phospholipid peroxides (PL-OOH) (146). Intracellular Fe²^+^ generates reactive oxygen species (·OH) via the Fenton reaction, further amplifying lipid peroxidation (147). When System Xc^−^ transporter function is inhibited, reduced cystein uptake limits GSH synthesis, preventing GPX4 from reducing PL-OOH and ultimately triggering ferroptosis (148). Although cells can provide alternative antioxidant mechanisms via the FSP1–CoQ10 and GCH1–BH4 pathways, these remain insufficient to prevent lipid membrane damage under high oxidative stress (149, 150). Iron metabolism-related proteins (TFR1, FTH1, FPN) and transcription factors p53 and NRF2 also participate in iron homeostasis regulation (151, 152).

Interferon plays a pivotal role in regulating ferroptosis. IFN-γ downregulates the expression of System Xc^−^ subunits SLC3A2 (solute carrier family 3 member 2) and SLC7A11 (solute carrier family 7 member 11) via the JAK/STAT1 pathway, inhibiting cystine uptake. This leads to glutathione (GSH) depletion, GPX4 inactivation, and lipid peroxidation accumulation, thereby inducing ferroptosis in tumor cells (153). This mechanism significantly enhances tumor sensitivity to immunotherapy and correlates closely with anti-PD-1 efficacy (154). Ferroptosis also releases DAMPs, activating dendritic cells and promoting cross-presentation of antigens, thereby enhancing CTL and NK cell function (155). Furthermore, ferroptosis-derived lipid peroxidation products can induce IFN-I responses via the cGAS–STING pathway, promoting dendritic cell maturation and T cell expansion, thereby amplifying antitumor immune effects (156). Preclinical studies demonstrate that ferroptosis inducers combined with immune checkpoint inhibitors significantly enhance immune responses and tumor suppression, providing a novel theoretical basis for combined immunotherapy and metabolic targeting strategies (157, 158).

## 4 Gut microbiota modulates interferon-induced programmed cell death in colorectal cancer

The gut microbiota, interferon, and programmed cell death collectively form a core network sustaining intestinal immune homeostasis. Under healthy conditions, this axis achieves a balance between host defense and tissue repair by the microbiota positively regulating interferon production, while interferon induces moderate PCD to maintain barrier renewal and microbial diversity (21). However, during colorectal cancer development, this network progressively dysregulates, transforming from a protective mechanism into a key driver of immune evasion and tumor progression (17). Microbiota modulate interferon signaling. The commensal microbiota activates interferon expression from diverse sources through molecular patterns and metabolic products. Macrophages and plasmacytoid dendritic cells (pDCs) secrete IFN-I in response to microbial stimulation, while intestinal epithelial cells IFN-λ. These signals are essential for initiating and sustaining natural killer (NK) cell and CD8^+^ T cell function. Furthermore, pDC-derived IFN-I enhances antigen presentation capacity in conventional dendritic cells (cDCs) through epigenetic reprogramming (21, 159).

### 4.1 Gut microbiota modulates type I interferon-induced apoptosis in colorectal cancer

In colorectal cancer, IFN-I bridge innate immune recognition with tumor cell apoptosis. Cytoplasmic DNA fragments induced by radiotherapy or chemotherapy can trigger IFN-I expression via the cGAS–STING pathway. Microbiota-associated molecules, such as bacterial DNA and cyclic dinucleotide c-di-AMP, can also enhance this signaling through the same pathway, and microbiota-derived molecules can amplify IFN-I signaling via this pathway. Poly-γ-glutamic acid from Bacillus species, Bacteroides fragilis polysaccharide A (PSA), and Lactobacillus nucleic acids can all induce IFN production via TLR4, RIG-I-like receptors (RLRs), or the cGAS pathway (78, 160–163). Metabolites are equally important: SCFAs produced by the family Clostridiaceae enhance IFN-I expression via GPR43 (164); secondary bile acids converted by Clostridium species induce pDCs to secrete IFN-I (165).

**Figure 1:**
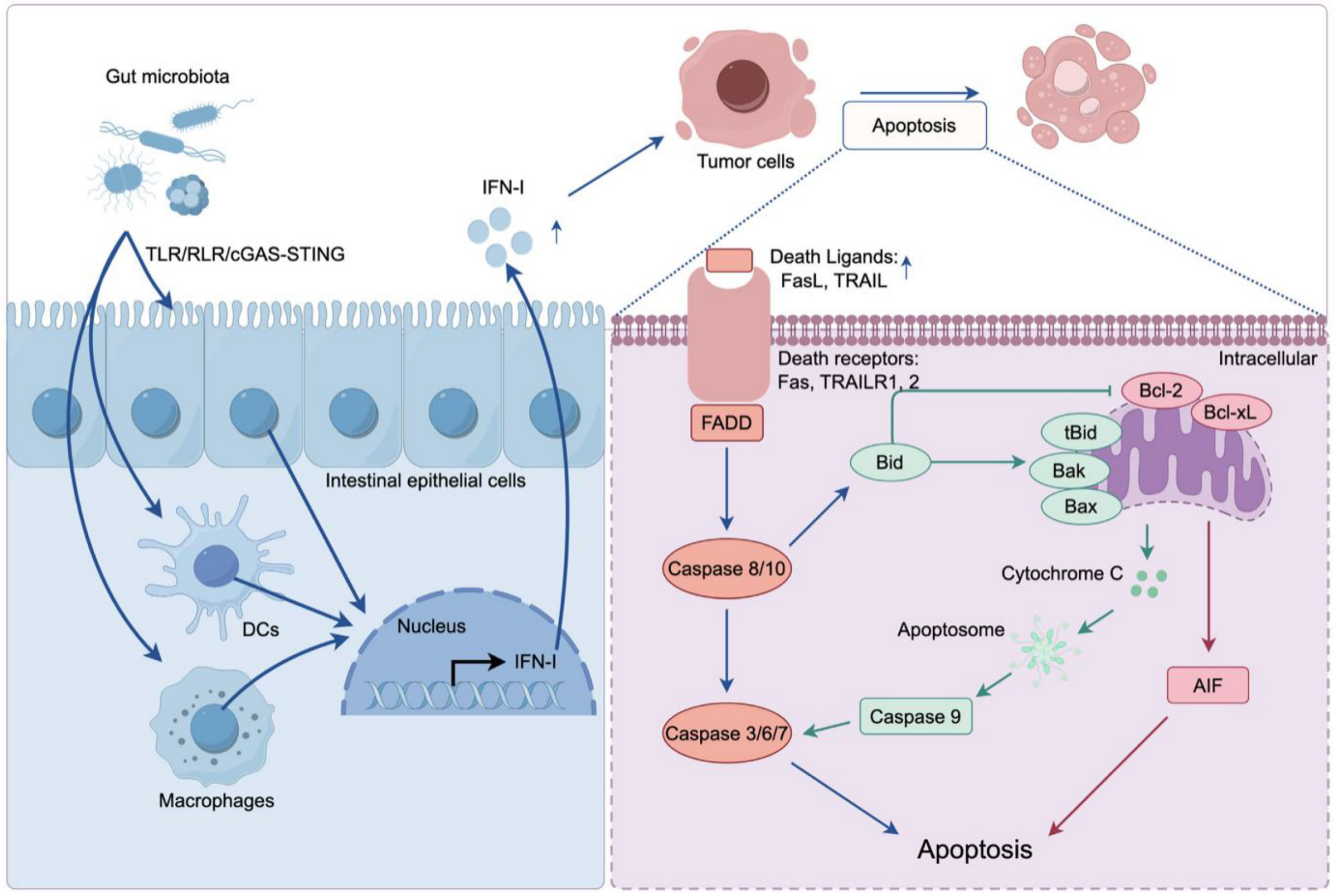
Gut microbiota promotes interferon-induced tumor apoptosis. Gut microbiota–derived microbial signals activate pattern-recognition receptors, including TLRs, RLRs, and the cGAS–STING pathway, in intestinal epithelial cells, dendritic cells (DCs), and macrophages. Engagement of these innate immune sensors drives robust production of IFN-I. IFN-I upregulates death ligands (FasL, TRAIL) and enhances their engagement with death receptors on tumor cells, triggering FADD-mediated activation of caspases-8/10 and downstream effector caspases-3/6/7. Caspase-8–generated tBid activates Bax/Bak, inducing mitochondrial permeabilization and the release of cytochrome c and AIF. Cytochrome c promotes apoptosome formation and caspase-9 activation, whereas AIF facilitates chromatin condensation. Anti-apoptotic Bcl-2/Bcl-xL counteract this process. Together, extrinsic and intrinsic pathways converge to promote IFN-I–dependent tumor cell apoptosis.

At the level of tumor immune surveillance, IFN-I induce programmed tumor cell apoptosis by upregulating TRAIL and its receptors and activating caspase-8 (166, 167); promoting mitochondrial depolarization, decreasing Bcl-xL while increasing Bim and PARP-associated signals with AIF translocation (168), and enhancing DC maturation, antigen cross-presentation, and CD8^+^ T-cell expansion (169). IFN-I further upregulates tumor-cell MHC-I expression (170, 171). It suppresses anti-apoptotic proteins Bcl-2 and Bcl-XL (172, 173) through Jak/STAT signaling, while synergizing with TRAIL via ERK signaling to promote apoptosis (**Figure 1**) (174). Except for apoptosis, IFN-I intersects with additional programmed cell death pathways in colorectal cancer. In ferroptosis, cGAS–STING–induced IFN-β increases intracellular Fe²^+^ and lipid peroxidation, lowers glutathione, and upregulates TRIM22, thereby amplifying RSL3-induced ferroptosis in HCT116 and other tumor cells (175, 176). In pyroptosis, IFN-I–induced guanylate-binding proteins (GBPs) facilitate caspase-11-dependent noncanonical inflammasome activation and GSDMD-mediated lytic death, and IFN-I signaling is required for full activation of the NLRP3 inflammasome (138, 177). Collectively, these findings indicate that gut microbiota–modulated IFN-I signaling integrates innate immune sensing with multiple programmed cell death modalities—including apoptosis, ferroptosis, and pyroptosis—to shape antitumor immunity in colorectal cancer.

### 4.2 Gut microbiota modulates type II interferon-induced programmed cell death in colorectal cancer

In the colorectal cancer microenvironment, tumor-infiltrating effector T cells and natural killer cells are the primary sources of IFN-γ, with secondary sources including Foxp3^+^ CD4^+^ regulatory T cells (Tregs), Th17 cells, Th22 cells, NKT cells, innate lymphoid cells (ILCs), and certain antigen-presenting cells (178). Microbiome homeostasis significantly impacts IFN-γ function: for instance, enrichment of Akkermansia muciniphila enhances IFN-γ^+^ CD8^+^ T cell infiltration and markedly improves PD-1 blockade efficacy; colon IFN-γ production increases substantially upon Gram-negative bacterial exposure, a process dependent on monocytes and myeloid dendritic cells (179). Furthermore, microbial metabolites such as SCFAs improve metabolic adaptability in CD8^+^ T cells and enhance their IFN-γ secretion capacity, thereby promoting ferroptosis and pyroptosis (180). They also influence IFN-γ production by regulating macrophage polarization (181). Conversely, dysbiosis or pathogenic bacteria (e.g., pks-carrying E. coli) weaken the IFN-γ–PD-1 pathway by releasing immunosuppressive factors, leading to immune tolerance and drug resistance (182, 183).

**Figure 2:**
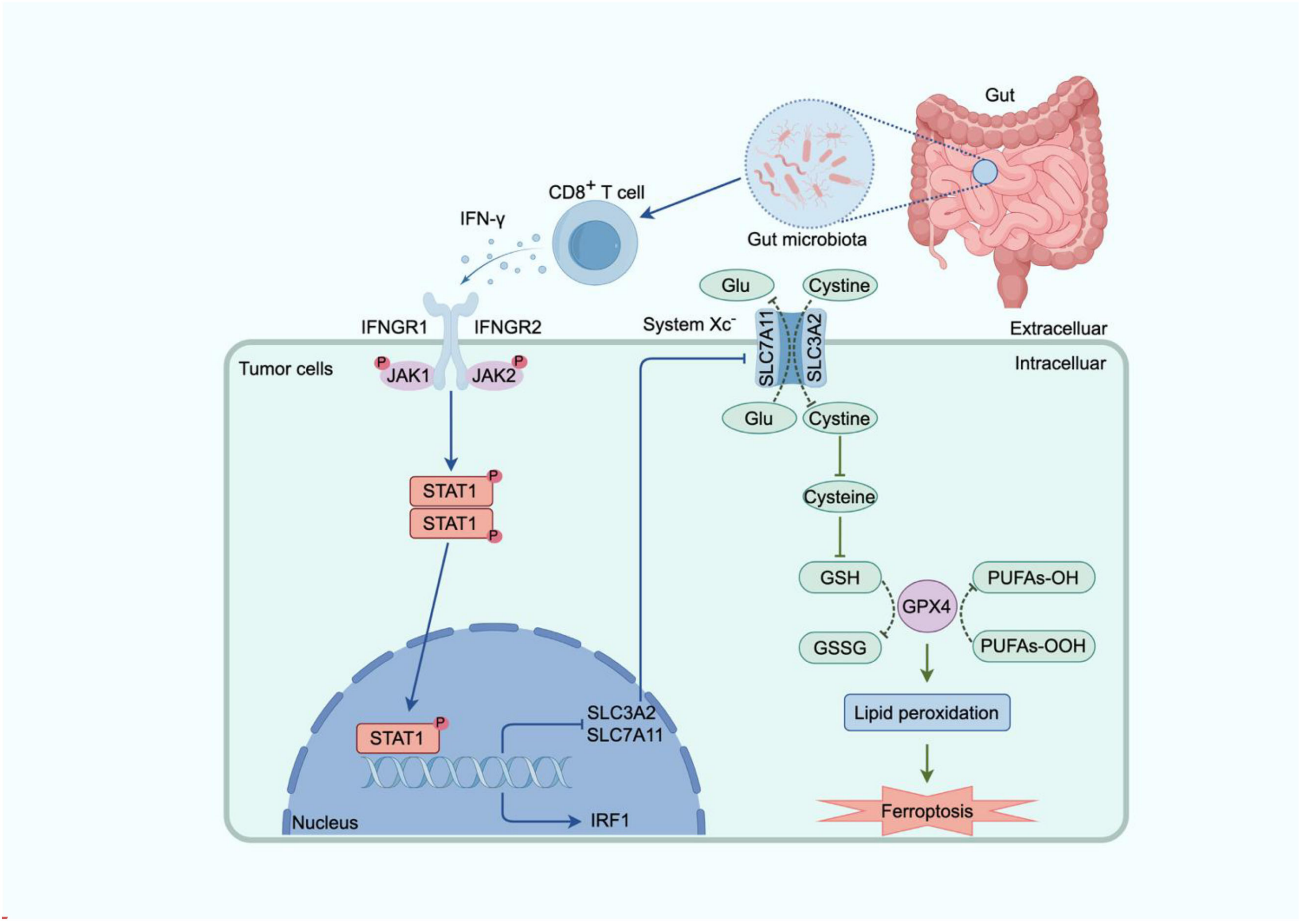
Molecular mechanism by which IFN-γ promotes ferroptosis in tumor cells. Gut microbiota–derived signals activate host immune pathways and promote CD8^+^ T-cell secretion of IFN-γ. IFN-γ engages the IFNGR1/IFNGR2 complex on tumor cells, triggering JAK1/2-mediated phosphorylation and activation of STAT1. Nuclear STAT1 induces IRF1, which transcriptionally represses the System Xc^−^ components SLC7A11 and SLC3A2, thereby reducing cystine import and limiting GSH synthesis. GSH depletion decreases GPX4 activity, allowing lipid peroxides to accumulate. The resulting lipid peroxidation compromises membrane integrity and drives ferroptotic tumor cell death.

IFN-γ exerts multifaceted roles in antitumor immunity. It upregulates MHC-I/II expression in tumor cells and APCs, enhances antigen presentation, and promotes APC secretion of IL-12, thereby driving Th1 polarization. Concurrently, IFN-γ induces Th1-type chemokine production, facilitating tumor infiltration by T cells and NK cells. Additionally, IFN-γ directly inhibits tumor cell proliferation (184–186) and induces cancer cell apoptosis (180, 187, 188) and necrotic cell death (189) via mitochondrial pathways. Recent studies further reveal that IFN-γ drives ferroptosis by inhibiting System Xc^−^, reducing glutathione synthesis, and weakening GPX4 antioxidant defense—a mechanism critical for immune checkpoint inhibitor (ICI) efficacy (190). Ferroptosis serves as a critical determinant of tumor progression and clinical prognosis in colorectal cancer, with its associated gene expression closely correlated with patient survival—even outperforming traditional TNM staging in prognostic assessment (191, 192). Recent studies indicate that ferroptosis sensitivity is regulated by metabolic reprogramming and the mTORC1 signaling pathway. The combination of aspirin and RSL3 effectively induces ferroptosis in PIK3CA-mutant CRC cells by inhibiting the mTOR/SREBP-1/SCD1 axis (193). In-depth elucidation of its molecular mechanisms, particularly the identification of key ferroptosis-associated biomarkers, will provide novel insights and strategies for early diagnosis and personalized treatment of colorectal cancer. (**Figure 2**)

### 4.3 Gut microbiota modulates type III interferon-induced apoptosis in colorectal cancer

In colorectal cancer, IFN-III primarily act on intestinal epithelial cells, serving as “guardians” maintaining mucosal barrier homeostasis. Their main sources include intestinal epithelial cells themselves, mucosa-associated dendritic cells, and neutrophils under specific infectious conditions (194). Gut symbiotic microbiota (e.g., Enterobacteriaceae) can weaken mucosal defense by downregulating the IFN-III response, thereby promoting carcinogenic inflammatory environments. Conversely, antibiotic or probiotic interventions can partially restore IFN-λ signaling and improve immune homeostasis (195). Secondary bile acids converted by Clostridium species stimulate epithelial cells to secrete IFN-λ (196).

Under intestinal homeostasis, commensal microbiota induce localized IFN-λ–ISG responses in the small and large intestinal epithelium, whereas such tonic signaling is markedly diminished in germ-free or antibiotic-treated animals and can be restored upon microbial re-colonization, indicating that the microbiota are essential for maintaining baseline epithelial IFN-λ activation (197). In the tumor context, IFN-λ activates IFNLR1-dependent caspase-3/8/9 signaling to induce G1/G0 cell-cycle arrest and apoptosis in colorectal cancer cells, exhibiting stronger cytotoxicity than IFN-I or IFN-II (198), consistent *in vitro* and *in vivo* evidence that recombinant IFN-λ1 suppresses tumor growth in a dose-dependent manner (199). Moreover, IFN-λ upregulates ZBP1 and promotes caspase-8–mediated GSDMC cleavage, thereby inducing pyroptosis-like lytic cell death in intestinal epithelial cells (119). Although there is a lack of direct evidence that IFN-λ triggers ferroptosis or necroptosis-like cell death in CRC cells, its signaling intersects extensively with oxidative stress, inflammatory, and metabolic pathways, and its regulation of inflammasome- and cytokine-related genes suggests potential interactions with additional regulated cell death (RCD) programs (197, 200, 201). Given its role in restraining NLRP6/NLRP9b inflammasome activation and epithelial IFN-λ secretion, and its emerging involvement in PERK-dependent ER stress–induced apoptosis,TRIM29 dysregulation may constitute a critical molecular hub linking impaired mucosal immune homeostasis, increased susceptibility to enteric viral infection, attenuation of IFN-λ–dependent tumor-suppressive signaling, and inflammation-driven colorectal carcinogenesis (202–205).

## 5 Summary and outlook

This review provides a comprehensive overview of the multifaceted interactions and mechanisms among the gut microbiota, IFN signaling pathways, and PCD within the immune microenvironment of CRC. IFNs serve not only as a pivotal bridge linking innate and adaptive immunity but also influence tumor cell survival and immune response states by finely regulating multiple PCD modes, including apoptosis, autophagy, pyroptosis, and ferroptosis. As key upstream regulators, gut microbiota modulate IFN function through metabolites (e.g., short-chain fatty acids), microbial-associated molecular patterns (e.g., LPS), and immune signaling molecules. In CRC, “protective microbiota” enhances IFN signaling and promote immunogenic cell death, activating the DC–CD8^+^ T cell axis; conversely, “oncogenic microbiota” suppresses IFN responses, disrupt epithelial barriers, and recruit immunosuppressive cells, facilitating immune evasion and drug resistance.

Several critical questions remain in this field. The highly context-dependent and cell-type-specific nature of the microbiota–IFN–PCD axis, along with its role in different CRC molecular subtypes, spatial heterogeneity, and dynamic evolution, remains unclear. Techniques such as single-cell multi-omics and spatial transcriptomics hold promise for revealing cellular interaction networks at higher resolution (206, 207). Most mechanistic evidence remains derived from preclinical models, requiring large-scale clinical validation for human applicability and individual variability. Furthermore, the dual role of type III IFN in mucosal immunity and epithelial repair necessitates more precise regulatory strategies.

Intervention strategies may offer novel avenues for CRC immunotherapy. Dietary modulation, probiotic supplementation, or fecal microbiota transplantation (FMT) can reshape “immune-supportive” microbiota to enhance IFN signaling and anti-tumor PCD (208, 209). Multimodal therapies targeting IFN pathways (e.g., IFN-γ combined with immune checkpoint blockade) or downstream PCD effector molecules (e.g., ferroptosis inducers) may overcome treatment bottlenecks in pMMR/MSS-type CRC (207, 210, 211). An individualized immune scoring system integrating multi-omics data will advance precision classification and treatment of CRC (206), positioning the “microbiota–IFN–PCD” axis as a novel target for clinical translation and drug development.
